# Multi-Criteria Optimization including Environmental Impacts of a Microwave-Assisted Extraction of Polyphenols and Comparison with an Ultrasound-Assisted Extraction Process

**DOI:** 10.3390/foods12091750

**Published:** 2023-04-23

**Authors:** Alice Bouchez, Peggy Vauchel, Sandrine Périno, Krasimir Dimitrov

**Affiliations:** 1 UMR-T 1158, BioEcoAgro Univ. Lille, INRAE, Univ. Artois, Univ. Littoral Côte d’Opale, JUNIA, Univ. Liège, Univ. Picardie Jules Verne, Institut Charles Viollette, F-59000 Lille, France; bouchez.alice@gmail.com (A.B.); peggy.vauchel@univ-lille.fr (P.V.); 2 GREEN Extraction Team, UMR 408, Avignon University, INRAE, F-84000 Avignon, France

**Keywords:** microwave-assisted extraction, multi-criteria optimization, polyphenols, antioxidants, by-product, beet seeds, LCA, environmental impacts, energy consumption, ultrasound-assisted extraction

## Abstract

Valorization of wastes and by-products using environmentally friendly technologies with an optimal cost–benefit relationship is a current major issue in agri-food industries. An original tool was recently developed for multi-criteria optimization of an ultrasound-assisted extraction (UAE) process including the assessment of environmental impacts using Life Cycle Assessment. In the present work, this methodology was adapted and applied to another green extraction process, microwave-assisted extraction (MAE), with the same case study, valorization of antioxidant polyphenols from downgraded beet seeds. Once built, the obtained multi-criteria optimization tool was used to investigate performances of the MAE process regarding productivity criteria (polyphenol concentration and antioxidant activity of the extracts), energy consumption and environmental impacts as functions of operating parameters (time, solvent composition, microwave power density, and liquid–solid ratio). The MAE process was optimized under different constraints and compared to the UAE process. For the studied conditions and different investigated scenarios, MAE enabled obtaining extracts with higher polyphenol concentrations and antioxidant activity (approximately 33% and 23% enhancements, respectively), and to strongly reduce extraction duration (by a factor up to 6), whereas UAE enabled reducing the energy consumption (up to 3.6 fold) and the environmental impacts (up to 12% for climate change).

## 1. Introduction

One important challenge in agri-food industries is the exploitation of their derived wastes and by-products as sources of new products using environmentally friendly technologies with an optimal cost–benefit relationship [1]. A wide range of biomolecules of interest, such as polyphenols, proteins, lipids, and vitamins, could be extracted from such vegetal matrixes providing better valorization. For example, polyphenol-rich extracts could be used as natural antioxidants in the food and cosmetic industries. On the other hand, the solid residues after polyphenol extraction could present higher carbon contents and calorific values than the untreated by-products, enabling multiple valorization of vegetal sources, in agreement with biorefinery goals [2].

To extract the target biomolecules using less energy and solvent than conventional extraction processes and with a reduction in risks, several alternative green processes have been developed [3]. Green extractions include microwave-assisted extraction (MAE), ultrasound-assisted extraction (UAE), electrically assisted extractions, pressurized liquid extraction, and supercritical fluid extraction, among others.

Microwave (MW) electromagnetic radiation induces a molecular dipole rotation, resulting in a very rapid dielectric heating of the polar components present in the vegetal matrix and in the solvent used, leading to the improvement of the extraction kinetics and to the reduction in extraction duration and, therefore, energy consumption. In the last two decades, MAE has been widely applied for the extraction of biomolecules from various vegetal sources [4]. Recently, interest in using this process for the extraction of polyphenols from agri-food wastes and by-products has grown [5]. High efficiencies of MAE have been reported for polyphenol extraction from apple skin [2], banana peels [6], avocado peels [7], peach waste [8], spent coffee grounds [9], and cocoa bean shell [10].

Although MAE is generally considered as a green process, there is very little information about the real energy saving due to the microwave assistance and about the environmental impacts of the process. Life Cycle Assessment (LCA) is a tool that enables the quantification of potential environmental impacts, taking into account all the activities associated, from the raw materials to end-of-life processing [11,12]. Recently, LCA has been applied for the assessment of the environmental impacts of UAE and conventional extraction of polyphenols from chicory grounds [13] and caffeine from guarana seeds [14], of conventional extraction and pressurized liquid extraction of polyphenols from grape pomace [15], of conventional extraction of quercetin and fructooligosaccharides from onion wastes [16], and of MAE of pectin from orange peels [17]. To our knowledge, there are not existing studies of LCA applied on MAE of polyphenols.

Recently, an original methodology has been developed for multi-criteria optimization of an extraction process including the assessment of environmental impacts by LCA [18]. The process considered was UAE of antioxidant polyphenols from sugar beet by-products. It was the first model enabling prediction of the environmental impacts of the process, as well as the energy consumption, together with antioxidant activity and extraction yield in the function of operating parameters (ultrasound power density, liquid/solid ratio, and solvent composition). The modelling procedure described was thought to be applied to other processes when it was possible to describe mathematically the kinetics of the criteria of interest as functions of input parameters (the operating conditions).

The objective of the present study was to adapt and to apply this multi-criteria optimization model to MAE of polyphenols from the same agri-food by-product: downgraded beet seeds. The MAE process will be optimized under different constraints and the obtained extraction yields, the antioxidant activities of the extracts, the energy consumption and the environmental impacts of the MAE process will be compared to those obtained in the case of UAE of polyphenols from this by-product.

## 2. Materials and Methods

### 2.1. Origin and Pretreatments of Beet Seeds By-Product

The sugar beet seeds used in present work consisted of the seeds rejected at the sort stage in SAS Vanderhave (Tienen, Belgium) because of some quality criteria (size, germination capacity, etc.) (same by-product lot as in the previous work [18]). The untreated seeds were entire seeds with a spherical shape and a diameter in the range of 4–6 mm. Two pretreatments were applied: crushing and powder grinding. Crushing was performed with a manual grain grinder mill to obtain large pieces of seeds (particle size of 2–4 mm). Powder grinding was carried out with a classic kitchen electric coffee grinder. A double blade was rotated at high speed until the seeds were reduced into powder (particle size < 0.5 mm).

### 2.2. Analytical Methods

Total phenolic compound concentrations were quantified according to Singleton et al. [19] and expressed as g.L^−1^ GAE (gallic acid equivalent) using a calibration curve of gallic acid (>98% purity, Sigma-Aldrich, Saint-Quentin-Fallavier, France). Antioxidant activity was evaluated with the protocol described by Brand-Williams et al. [20] and expressed as mM TEAC (Trolox equivalent) using a calibration curve of Trolox (6-hydroxy-2,5,7,8-tetramethylchroman-2-carboxylic acid), Sigma-Aldrich (Saint-Quentin-Fallavier, France).

### 2.3. Study of Beet Seed Pretreatment Effect on the Extraction of Antioxidant Polyphenols

The effect of beet seed pretreatment was studied performing decoction and MAE experiments with entire, crushed and powdered beet seeds. All experiments were carried out in a 1 L glass boiling flask with 600 mL of a 50/50% (Vol.) ethanol–water mixture as solvent (ethanol (CWR France, >99% purity) and deionized water) and 30 g of seeds (entire, crushed or powdered), corresponding to a 20 mL.g^−1^ hydromodule (solvent volume/seed mass ratio). For decoctions, the glass boiling flask was placed in a flask heater and connected to a condenser with tap water circulation. MAE experiments were carried out in triplicate in an ETHOS X oven (Milestone, Italy), equipped with 2 magnetrons (generating microwaves at a power of up to 1900 W and a frequency of 2.45 GHz) and a rotating diffuser, distributing microwaves throughout the cavity. The glass boiling flask was placed in the oven and connected to a condenser with a water cooling system set at a temperature of 8 °C (Smart H150-2100 chiller, Lab Tech, Boston MA, USA). Microwaves were applied at a power of 500 W (corresponding to 833 W.L^−1^). The mixture was agitated by a magnetic bar stirring system included in the oven. All experiments started at room temperature (24 ± 2 °C), and then the temperature was not regulated: the solvent was heated to boiling and vapors were continuously condensed, so that, except during the initial heating phase, the temperature was close to the solvent boiling point (approximately 94 °C). Vapors were continuously condensed by the condenser and water cooling system, so as to maintain a constant solvent volume during extraction processes. An ETHOS X compact terminal enabled controlling the applied power and agitation, and monitoring the temperature during assays (by an infrared temperature sensor). For each assay, extraction kinetics was followed taking samples at 5, 10, 15, 30, 45, 60, 90 and 120 min.

Collected samples were centrifuged at 4700 rpm for 10 min (Heraeus Multifuge FR centrifuge, Thermo Fisher Scientific, Bourgoin, France) in order to remove solid residuals from the liquid extract before antioxidant activity (AA) and total polyphenol (TP) measurements.

### 2.4. Optimization of MAE of Antioxidant Polyphenols: Data Collection and Modelling

The modelling procedure for the optimization of UAE from beet seeds previously developed and described in detail by Bouchez et al. [18] was adapted and applied in the present work in order to obtain a multi-criteria optimization model for the studied MAE process. Briefly, it consisted of experimental data collection and modelling of energy consumption, antioxidant activity, polyphenol concentration, and environmental impact outputs (using LCA methodology).

#### 2.4.1. Data Collection

MAE data were collected on the basis of experimental runs performed under different conditions according to a central composite design (CCD, Box–Wilson design) extended to the external cube domain. Three operating parameters were studied: ethanol content in the solvent (*X_1_*, %Vol.), liquid/solid ratio (*X_2_*, mL.g^−1^) and microwave power density (*X_3_*, W.L^−1^). The parameters were varied at five coded levels: −1.68, −1, 0, +1 and +1.68. Real values were in the following ranges: 0–100%Vol. for ethanol content, 5–35 mL.g^−1^ for the hydromodule, and 0–1667 W.L^−1^ for MW power density. A validation assay (supplementary assay for model validation) was performed under the following conditions: *X_1_* = 48%Vol., *X_2_* = 35 mL.g^−1^, and *X_3_* = 1027 W.L^−1^ (corresponding coded values: −0.07, +1.68, +0.39).

MAE assays were performed in the ETHOS X oven (Milestone, Italy) described above (Section 2.3), with the same equipment (magnetrons, magnetic stirring system, condenser and water cooling system set at 8 °C).

All assays were performed with crushed beet seeds. The solvent volume was 600 mL for all experiments and seed mass varied from 120 to 17 g, so as to obtain hydromodules between 5 and 35 mL.g^−1^. As detailed in Section 2.3, all assays started at room temperature (24 ± 2 °C); and after the initial heating phase, temperature was stable at the solvent boiling point (depending on the solvent composition). Electricity consumption of the whole experimental setup was monitored during each assay using an electrical consumption controller (Otio, France). For each assay, extraction kinetics was followed by regular sampling (at 5, 10, 15, 30, 45, 60, 90 and 120 min). Collected samples were centrifuged to recover a clarified liquid extract before TP and AA measurements.

For each experimental run and each sampling time, the output parameters measured were total polyphenols concentration (TP), antioxidant activity of the extract (AA) and energy consumption (EC).

#### 2.4.2. Modelling of Energy Consumption Output

Energy consumption at different operating conditions was expressed as a function of time considering electric power consumed by the stirrer and by the water chiller (same consumption whatever the values of *X_1_*, *X_2_*, and *X_3_*), and the electric power consumed for MW generation (depending on *X_3_* value). Energy consumed with centrifugation was constant regardless of parameter values. The corresponding value was added as a constant term to the previous function.

#### 2.4.3. Modelling of Polyphenols concentration and Antioxidant Activity of the Extracts

Empiric equations based on the Patricelli model [21] were used to describe the kinetics of antioxidant polyphenols extraction (the same type of equations were used for the total polyphenol concentration and for the antioxidant activity of the obtained extracts). The coefficients of these equations were expressed as functions of operating parameters *X_1_*, *X_2_*, and *X_3_*.

#### 2.4.4. Modelling of Environmental Impact Outputs Using LCA Methodology

LCA was performed in agreement with ISO standards [11,12], i.e., by following the four classic steps: goal and scope definition, life cycle inventory, life cycle impact assessment and life cycle interpretation. The details of the three first steps will be given in the following paragraphs and interpretation will be developed in the Results and Discussion section.

- *Goal and Scope Definition*

**Figure 1:**
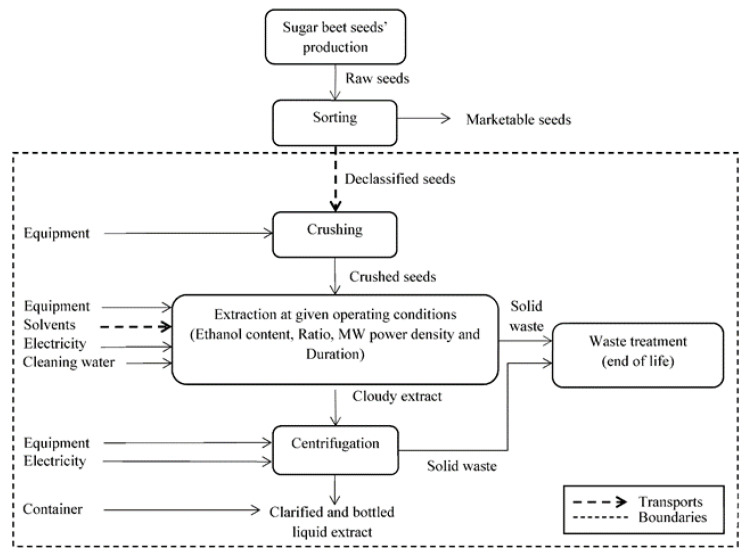
Boundaries of studied system for LCA.

Boundaries of the studied system are presented in Figure 1. They included all the steps from declassified beet seed recovery (after industrial sorting process) to the obtaining of a bottled liquid extract. They took into account equipment (raw materials, manufacture, dismantlement and end of life), solvent production and transport, seeds transport and crushing, the set of energy and water consumption due to extraction and centrifugation steps and the end of life of the residual material. The geographical scope was France.

Fields of study considered were the same for UAE and MAE to ensure that their impacts assessments were comparable under the condition of having set the same functional unit (FU).

- *Inventory*

Life cycle inventory was made with the help of Simapro software (version 9) associated with Ecoinvent 3 (at point of substitution, APOS), ELCD and Industry databases. The data are generic, experimental or calculated. The inventory is shared between five main stages: raw materials, transports, equipment, extraction stage, and end of life. The detail of elements considered for each stage is given in the following paragraphs.

The equipment data inventory included their complete life cycle from the raw materials required for their manufacture as well as the manufacturing processes and the corresponding end-of-life treatments. The modelling of each piece of equipment was carried out by weighing some items, estimating others using diagrams or pictures provided by the manufacturers. Once the equipment was modelled as accurately as possible, only a portion of the total lifetime of equipment was actually considered, in proportion to the duration of use (mass allocation method).

The transport included within the boundaries of the study was that of declassified seeds and ethanol. For the seeds, the distance travelled with a car between the sorting site and the laboratory was 170 km or a 340 km round trip. An allocation of impacts was then calculated for each extraction based on the introduced seed mass depending on the value of the ratio parameter. The ethanol was delivered by an external supplier (VWR) and the means of transport used was by default a lorry. Non-specific data on the type and size of truck were used. These data included an estimate of the conventional loading of a truck and allocated a share of the impacts to the mass of ethanol multiplied by the distance travelled. The distance travelled between the VWR site and the laboratory was 375 km or 750 km round trip. The mass of ethanol considered depended on the value of the ethanol content in the solvent used.

The raw materials used in the extraction process were crushed beet seeds and solvent. As the inclusion of seeds did not begin until they were taken on site after sorting, only the crushing process was accounted for as an impact related to the obtaining of the raw material “crushed seeds”. A crushing mill was modelled and an impact allocation was applied as for the other equipment. As the mill was completely manual, no energy consumption was considered by its use. The extraction solvent consisted of demineralized water and ethanol, pure or in a mixture. Petrochemical origin was considered for ethanol since, according to Garcia-Garcia et al. [17], ethanol produced from biomass would be mainly reserved for beverage or fuel production. Water was considered with generic data (water, deionized, from tap water).

The extraction stage implied an electric consumption needed for equipment, a water consumption for cleaning and the use of a container for the final extract. Electric consumption was monitored or calculated. Data used were those of French energy mix: electricity, low voltage {FR}. The breakdown of energy sources considered in these data was as follows: 78% nuclear, 11% hydro, 4% coal, 4% natural gas, 1% wind, 1% oil and 1% other sources [22]. The necessary amount of cleaning water was estimated to be 1 L (water, deionized, from tap water) with the end-of-life treatment of domestic waste water. The container was a glass bottle with a plastic cap.

The end-of-life stage refers only to the end of life of the process, that is to say the treatment of residual beet seeds after extraction. End-of-life treatments specific to some elements were directly included in the stages to which these elements belonged. It was considered that this waste was treated as classical household waste and therefore incinerated.

It should be mentioned that the analyses made in the present study were related to a lab-scale research and conditions (specifics of ethanol delivery, raw materials transportation, crushing method, MAE equipment, UAE equipment, centrifugation, liquid extract packaging, etc.).

- *Life Cycle Impact Assessment*

The environmental impacts were assessed with the ILCD2011 midpoint method [23]. With the use of this method, the impacts were shared between 16 categories: acidification, climate change, freshwater eco-toxicity, freshwater eutrophication, human toxicity cancer effects, human toxicity non-cancer effects, ionizing radiation ecosystem, ionizing radiation human health, land use, marine eutrophication, ozone depletion, particulate matter, photochemical ozone formation, terrestrial eutrophication, water resource depletion and mineral, fossil and renewable resource depletion.

## 3. Results and Discussion

### 3.1. Effect of Beet Seed Pretreatment on Polyphenols Extraction

**Figure 2:**
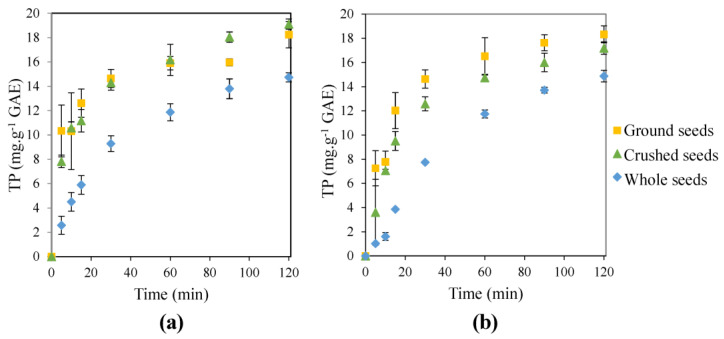
TP (expressed in yields) extraction kinetics for MAE (**a**) and decoctions (**b**) processes from whole, crushed or powder ground seeds (with ratio L/S: 20 mL.g^−1^, ethanol content: 50%(Vol.) and MW power density: 833 W.L^−1^).

Firstly, a preliminary study was carried out in order to elucidate the interest in pretreating the beet seeds before MAE studies. The kinetics of MAE of polyphenols from untreated seeds was compared to extraction kinetics from powdered and crushed seeds. The results given in Figure 2a show that both pretreatments had a clear positive effect on polyphenols extraction. The highest TP yields (19.1 mg GAE/g d.w.) were obtained in the case of crushed seeds (30% higher than for untreated seeds after 120 min). The kinetics of the extractions from pretreated seeds were clearly enhanced since the increase in the extraction yield from crushed seeds at 15 min was 135% higher; and at 30 min, 57% higher than for the untreated seeds. The extraction from powder ground seeds was the most rapid in the beginning of the extraction, after the first 15 min there was no real difference between MAE results obtained from powder ground and from crushed seeds. For further optimization studies of MAE, a crushing pretreatment was preferred for three reasons: the lower energy consumption, the easier separation of the liquid extract from exhausted seeds, and the possibility to compare the MAE results to those obtained in UAE from the same by-product: crushed beet seeds [18].

The comparison between the results in MAE (Figure 2a) and the results obtained using a conventional extraction at the boiling temperature (decoction, Figure 2b) shows the positive effect of MW assistance on the extraction, especially for the crushed seeds. In MAE the heating comes from the core of the extraction unlike decoction where the heating comes from to the outer wall. MW heating is faster, prevents overheating of the walls and a temperature gradient, and allows targeted heating of the products and then a better energy efficiency [24]. Only 4 min were necessary to reach the boiling temperature using MW, while 14 min were needed in decoction studies. After 10 min, yields were higher for MAE by 180% for whole seeds, 50% for crushed seeds and 36% for powder ground seeds. However, the results were quite similar after 15 min for powder ground seeds and 60 min for whole seeds.

### 3.2. Modelling of TP, AA, EC and EI Outputs for the Studied MAE Process

In order to obtain a multi-criteria optimization tool for the studied MAE process, it was necessary to build adapted and suitable models for TP, AA, EC and EI outputs as functions of input parameters (time, solvent composition, hydromodule, MW power density). Table 1 presents models established for MAE in the present study, with regard to those established for UAE in the previous study [18]. The main difference between the studied MAE and UAE processes relied on the existence of a “breaking time” in the case of the UAE process used, as the ultrasounds were applied in the continuous mode in the beginning of the experiments and after reaching 70 °C (corresponding time at this moment applied “breaking time”) they were applied in the discontinuous mode. For the studied MAE process, MW were applied always in the continuous mode so that breaking time was not applicable, thus modelling was quite simpler. TP and AA equations were setup on the base of the Patricelli equation [21], which appeared relevant to describe the extraction of phenolic compounds from sugar beet seeds both in the cases of UAE and MAE. These coefficients were expressed as second order polynomial functions of the coded values of the input parameters (solvent composition *X_1_*, liquid/solid ratio *X_2_* and MW power density *X_3_*).

EC was modelled as a linear function of time, taking into account energy consumption of the whole experimental setup, i.e., the MW generator, the water cooling system, the stirrer and the centrifugation.

All coefficients of model equations were found by minimizing the deviation between experimental kinetics data and model for all runs of the experimental design. This deviation was calculated with the NRMSD (Normalized Root Mean Square Deviation) statistical tool.

After the determination of all coefficients of the multi-criteria model of the MAE process, a validation experiment was carried out under conditions different to those used in the experimental design. The observed deviations between the model predictions and the experimental results of this validation experiment were satisfactory and the model was validated. For *EC*, the NRMSD value for the validation run was 1.5% (average NRMSD of the model for the 24 assays of 2.7%, minimal of 0.2%, maximal of 7.9%). The NRMSD value for *TP* for validation experiment was 7.6% which was close to the average NRMSD for *TP* (7.0%) and included in the variation range for the whole experimental set (2.1–18%). A similar NRMSD value (8.1%) was observed for *AA*, close to the average NRMSD for *AA* of 10% and included in the variation range 4.3–21%.

### 3.3. Multi-Criteria Optimization of MAE

#### 3.3.1. Maximization of Polyphenols Extraction and Antioxidant Activity of the Extracts

**Figure 3:**
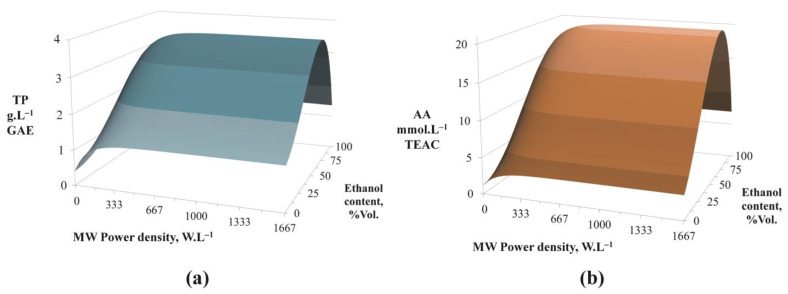
Predicted values for TP concentrations (**a**) and for AA of the extracts (**b**) in function of MW power density and ethanol content (for a ratio L/S of 5 mL.g^−1^ and a duration of 120 min).

**Figure 4:**
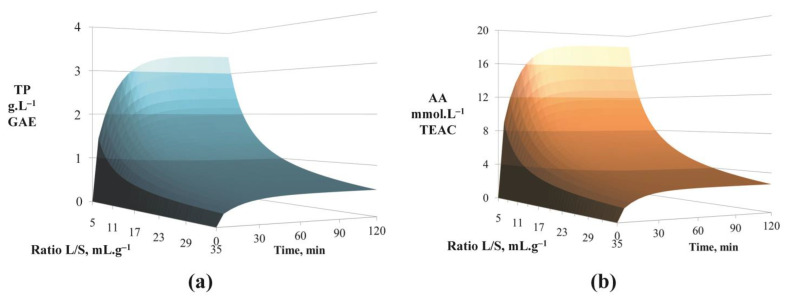
Predicted values for TP concentrations (**a**) and for AA of the extracts (**b**) in function of ratio L/S and time (for a MW power density of 1333 W.L^−1^ and an ethanol content of 50%Vol.).

In order to obtain a first overview of the effects of input parameters on *TP* and *AA* results, 3D graphs were created to represent the results calculated with the multi-criteria model for MAE (Figure 3 and Figure 4). Prior to any other observation, it was found that, in the same way as for UAE, the trends in *TP* concentrations and *AA* of the extracts obtained by MAE were correlated whatever the parameters studied. Moreover, MAE trends were very similar to those of UAE [18]. In fact, they had a maximum around the same value of ethanol content (approximately 65%Vol.) regardless of the power density applied (Figure 3). The increase MW power density had a positive impact on the extraction results. This effect was very pronounced for relatively small MW power densities but clearly lower beyond a certain value. For example, in Figure 3, it could be seen that plateaus were reached after a certain value of MW power density (approximately 300 W.L^−1^ for the solvent with an ethanol content of 65%Vol.) both for *TP* and *AA* outputs (for liquid/solid ratio of 5 mL.g^−1^ and a duration of 120 min).

Concerning the effect of the hydromodule, as expected, *TP* and *AA* increased as the ratio decreased and this effect was more pronounced for smaller ratios (Figure 4). MAE was very rapid. *TP* and *AA* concentrations tended to plateaus after approximately 30–40 min whatever the value of the hydromodule (for MW power density of 1333 W.L^−1^ and an ethanol content of 50%Vol.).

The optimal conditions for MAE obtained by the multi-criteria optimization model are given in Table 2. Once again, the strong correlation between *TP* concentration and *AA* of the obtained extracts from beet seeds was demonstrated. The conditions that enabled obtaining the highest activity of the extracts were almost the same as those to obtain the maximal concentration of polyphenols. The optimal ethanol content was, as presumed before with the 3D graph of Figure 3, approximately 65%Vol. Then, as expected, the smallest value for the hydromodule (5 mL.g^−1^) led to the highest *TP* and *AA* values. The optimal duration was the maximal one (120 min) but the MW power density level needed was high (1160–1200 W.L^−1^) but lower than the highest used in the experimental design (1667 W.L^−1^). These optimal values were in agreement with the one found by Périno et al. in several studies of MAE [25,26] of 1 W.g^−1^ total mass (mass of solvent and plant matrix). Indeed, the weight of the 600 mL of 65%Vol. ethanol solvent was 522 g and the added mass of seeds was approximately 120 g (due to the hydromodule value of 5 mL.g^−1^). This led to a power density in the range of 1.09–1.12 W.g^−1^ total mass. Finally, the *EC* for the two scenarios (maximal *TP* and maximal *AA*) was close, with a difference between the values lower than 1.5%. To finish, the maximal concentrations reached with MAE were higher than those obtained using UAE from the same by-product (2.8 g.L^−1^ GAE and 16.6 mmol.L^−1^ TEAC, respectively [18]). This could be explained with the higher power input and the higher maximal temperatures in the case of MAE (solvent boiling temperature) than for UAE (70 °C).

**Figure 5:**
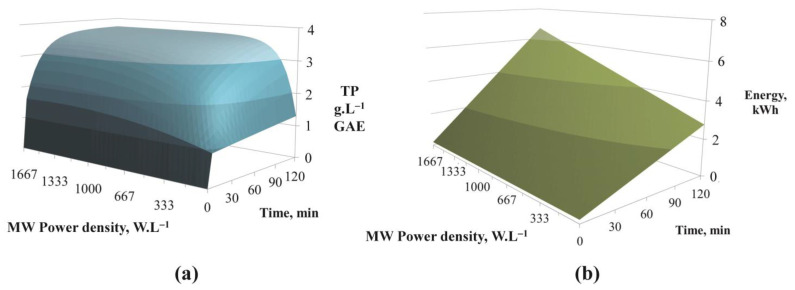
Predicted values for TP (**a**) and for EC (**b**) in function of MW power density and time (for a ratio L/S of 5 mL.g^−1^ and an ethanol content of 65%Vol.).

Despite the fact that it was the maximal duration that led to the maximization of *TP* and *AA* concentrations, it would still be interesting to observe whether it was really necessary to go up to 120 min of extraction to obtain interesting concentrations. In fact, the original multi-criteria optimization model used in this study enables correlating these outputs with the energy consumption and the environmental impact outputs. As an example, the 3D charts of *TP* and *EC* in function of time and MW power density for the optimal values of ethanol content and hydromodule found before (respectively 65%Vol. and 5 mL.g^−1^) are presented in Figure 5. It is clearly visible that beyond a certain MW power density value and a certain duration, the *TP* values varied very little (Figure 5a), while the *EC* continued to increase strongly with the latter two parameters (Figure 5b). For example, an amount of *TP* of 2.0 g.L^−1^ GAE could be achieved either by consuming 0.9 kWh (with a duration of approximately 25 min and a MW power density of 250 W.L^−1^) or by consuming 1.7 kWh (with 65 min and 83 W.L^−1^). This was partly due to the fact that the water cooler feeding the refrigerant ran continuously regardless of the MW power density value. There was therefore interest in crossing *TP* and *AA* data with the *EC* and *EI* generated.

**Figure 6:**
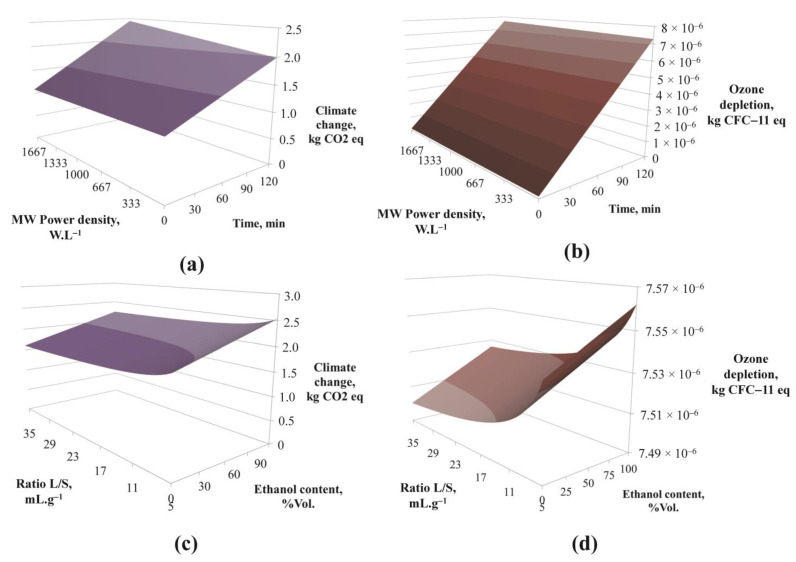
Predicted values for two categories of *EI* (climate change (**a**,**c**) and ozone depletion (**b**,**d**)) in function of MW power density and time ((**a**,**b**) for a ratio L/S of 5 mL.g^−1^ and an ethanol content of 65%Vol. and in function of ratio and ethanol content in the solvent; (**c**,**d**) for a MW power density of 1200 W.L^−1^ and a time of 120 min).

In the same way as for the *TP*, *AA* and *EC* results, it was possible to generate 3D charts for all of the *EI* categories in function of input parameters. To illustrate the effect of operating parameters on *EI*, the results for the categories “climate change” and “ozone depletion” are presented in Figure 6. These categories are considered at level I by the ILCD2011 midpoint method, thus they are less subject to uncertainty issues (less than 10% uncertainty for *EI* category I, up to 30% for *EI* category II, and higher for *EI* category III) [27,28]. Both *EI* increased with time, ethanol content in the solvent and MW power density, and decreased for higher ratios L/S (time and ratio effects more pronounced for “ozone depletion” category). These trends were representative of all *EI* categories.

#### 3.3.2. Multi-Criteria Optimization under Different Constraints

Hence, reducing the target *TP* or *AA* by a little could enable greatly reducing the different impacts (environmental or energetic consumption). The following paragraph therefore aimed at studying scenarios with targets lower than the achievable maxima. In order to compare different scenarios for MAE using different constraints, a FU was setup to 16.6 mM TEAC for the *AA* of the extracts. This value was the maximal one obtained in the case of UAE from the same by-product [18] and represented approximately 80% of the maximal one in the case of MAE (20.5 mM TEAC, Table 2).

Then, five scenarios were simulated with the model with the following constraints:

- Minimize energy consumption (*EC*);
- Minimize duration (*t*);
- Minimize the *EI* category “climate change” (*CC*);
- Minimize ethanol content in solvent (*X_1_*);
- Minimize MW power density applied (*X_3_*).

**Figure 7:**
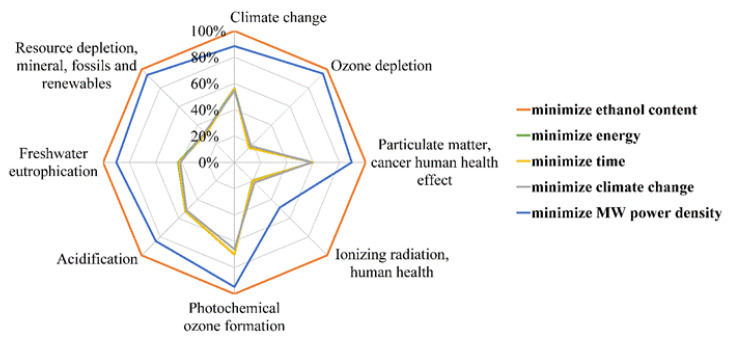
Kiviat chart for eight *EI* emitted by each of the scenarios presented in Table 3 (target objective: AA of 16.6 mM TEAC).

The model predictions for each scenario studied were reported in Table 3 for the input parameters and *EC* values. The *EI* were represented with a Kiviat chart, with each axis corresponding to one of the *EI* categories. Due to the variety of units of measurement for the *EI*, the values were expressed as a percentage of the maximal amount reached by one of the scenarios. This Kiviat chart corresponding to the scenarios described in Table 3 is presented in Figure 7. Within the 16 estimated *EI*, it was chosen to present the 8 *EI* recommended on Kiviat charts at level I and II by the ILCD2011 midpoint method, so that they are thus less subject to uncertainty issues [27].

Observing Table 3 and Figure 7, the scenarios which aimed to reduce *EC*, time or *CC* led to similar durations, *EC* and *EI*. In fact, in these three cases, duration was in the range 17–20 min, *EC* in the range 1.08–1.25 kWh and *EI* were overlaid on the chart. It was not possible to attain the target objective (16.6 mM TEAC) without ethanol in the solvent, nor without MW assistance. The scenario that aimed to reduce ethanol content led to a non-significant reduction in ethanol and a need for much more energy and time (approximately 6-fold more) compared to the scenario that aimed to minimize time using the same MW power density value. The scenario with the objective to minimize MW power density also implied very high *EC* and maximal duration of the extraction process. Most of the *EI* categories were also significantly higher for these last two scenarios. These high values of *EI* for the last two scenarios could be explained by the fact that they both required very long extraction times and consequently a large equipment mass allocation. In addition, since the cooling and MW generator devices were large consumers of electricity, keeping them switched on for long periods of time implied a high *EC*, which also increased most of the *EI*. Thus, ethanol content and MW power density had beneficial effects on the reduction in *EC* and *EI* of MAE when, simultaneously, ethanol content was close to its optimal value (approximately 66%) and MW power density was high (above 700 W).

### 3.4. Comparaison between MAE and UAE

The UAE and MAE processes were modelled with the same input parameters: duration, ethanol content, hydromodule, and power density of the assistance technology (US or MW). Consequently, it was possible to make comparisons between the two processes. For this purpose, a FU was chosen to be reachable by both of the two processes. The objective being to have an extract with the greatest *AA*, it was the maximal value reachable with UAE which was selected: 16.6 mM TEAC. The corresponding input parameters (reported in Table 4) were the optimal ones for UAE (determined in the previous study [18]). The parameters chosen for MAE to achieve this FU were simulated according to two constraints:

- Consume less electricity as possible (constraint n°1);
- Apply the same power density, ratio and ethanol content as UAE scenario with MAE, i.e., respectively 667 W.L^−1^, 5 mL.g^−1^ and 65%Vol. (constraint n°2).

Hence, three scenarios were simulated to reach the FU of an *AA* of 16.6 mM TEAC. The first one used UAE technology with a US power density of 667 W.L^−1^ and the extraction lasted 120 min with a switch from continuous to discontinuous mode of US emission at approximately 17 min. The two other scenarios used MAE with a MW power density of 1300 W.L^−1^ for 19 min to satisfy the first constraint (minimize *EC*) and 667 W.L^−1^ for 30 min to satisfy the second one (same value of power density as UAE scenario). Consequently, in both MAE scenarios, there was significant time saving compared to UAE. In contrast, *EC*, which was close for the two MAE scenarios, was much higher than in the UAE scenario (3.2 to 3.6 fold).

For the three scenarios, the ethanol contents required were very similar (65%Vol. or 66%Vol.). The hydromodule value of 5 mL.g^−1^ was expected because it is the one which implied adding the greatest quantity of seeds and, as mentioned previously (Figure 4), allowed the recovery of the extracts most concentrated in *TP* and *AA* for the two technologies.

**Figure 8:**
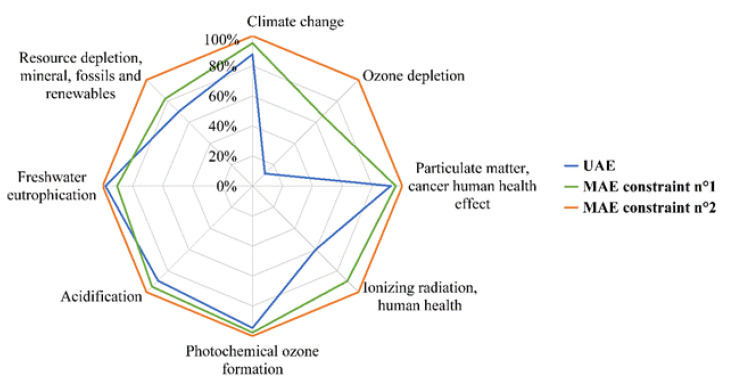
Kiviat chart for eight *EI* emitted by each of the scenarios presented in Table 4 (target objective: *AA* of 16.6 mM TEAC).

Observing the Kiviat chart in Figure 8 allowed a quick visualization of which scenario had the lowest *EI*. Based on the uncertainty values given in Section 3.3, the two MAE scenarios generated almost identical EI values (i.e., differences lower than significance thresholds) while the UAE scenario had significantly lower values for three categories of *EI*:

- Ozone depletion (up to 82% lower than MAE scenarios);
- Ionizing radiation human health (up to 35% lower than MAE scenarios);
- Climate change (up to 12% lower than MAE scenarios).

The other differences were considered as not significant.

**Figure 9:**
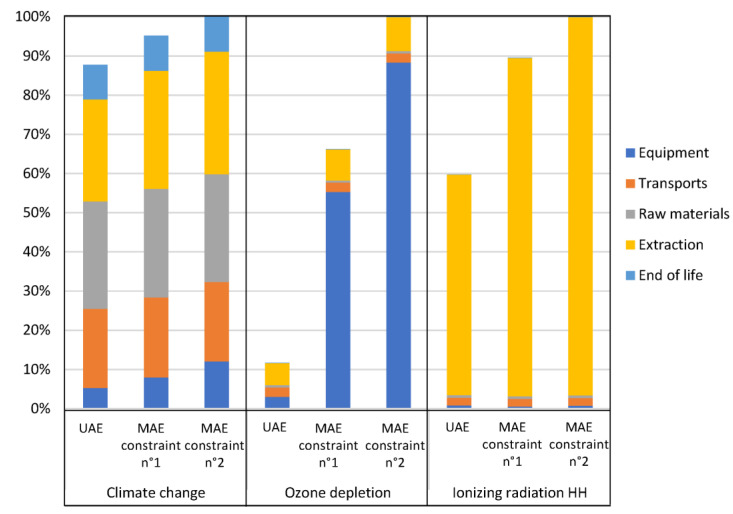
Comparison of the scenarios presented before (Table 4, target objective: AA of 16.6 mM TEAC) for three EI categories (climate change, ozone depletion and ionizing radiation human health (HH)) with distribution between life cycle stages.

In order to explain the significant differences, more details are given in Figure 9. On this bar chart, the values for the three categories mentioned above are represented for each scenario as a percentage of the highest value reached by one of the scenarios in each impact category with the distribution between the five life cycle stages (raw materials, equipment, transport, extraction and end of life). Consequently, for both the MAE scenarios, the high level reached by the *EI* category “ozone depletion” in comparison to the UAE scenario was mainly due to the equipment stage. This result could be surprising because the allocated equipment mass hardly changed, as the 6-fold longer extraction time for UAE counterbalanced the difference in mass of the UAE equipment approximately 6-fold lower than that of the MAE equipment. The difference was therefore due to differences in the materials considered in the equipment inventory. Apparently in the MAE equipment used in the present study there were some components from materials and in mass (namely the magnetrons) that caused higher impacts on “ozone depletion” comparing to materials in the UAE equipment used).

For the category of impact “ionizing radiation human health”, it was the extraction stage that accounted for the largest share of the impact and which varied between scenarios. This variation was proportional to the share of *EC* required by the extraction itself (without centrifugation), which was expected as it was the only variable data for the extraction stage of the three scenarios. The difference between UAE and MAE for “climate change” category resulted both from the differences between the extraction equipment used and the energy used in the extraction steps of these two processes.

Overall, in addition to consuming less electricity, UAE generated the lowest amount of *EI* to reach the defined FU. From an environmental point of view, it was therefore this process that should be favored. However, the time saving brought by the use of MAE was not negligible and, from the point of view of productivity, it is this process that should be chosen. MAE also enabled obtaining higher extraction yields and concentrations and higher antioxidant activity of the extracts.

## 4. Conclusions

MAE is an appropriate process for the extraction of antioxidant polyphenols from beet seed by-products. The original multi-criteria optimization model applied for the MAE process allowed considering the productivity criteria (polyphenols concentration and antioxidant activity of the extracts) together with the energy consumption and the environmental impacts of the process as functions of the operating parameters (time, solvent composition, MW power density, and the ratio between the solvent volume and the mass of the by-product). The simultaneous consideration of the effects of operating parameters on the four studied outputs enables determining the conditions that enable obtaining an extract with a target antioxidant activity (or target polyphenol concentration) using minimum energy or with the lowest environmental impacts. The comparison between the MAE and UAE processes used on the same by-product shows that MAE enables obtaining higher polyphenol concentrations and antioxidant activity of the extracts and strongly reducing the extraction duration. In contrast, the UAE process needs less energy and the environmental impacts of this process are lower than those in the case of MAE (for the studied experimental conditions and the respective equipment used). So, one could wonder if there would be an interest in combining these two technologies. Indeed, this combination could enable cumulating the advantages of each technology:

- The improvement of extraction at the cost of a small increase in *EC* by US assistance;
- The significant decrease in extraction duration due to MW assistance.

Hence, this combination seems promising and will be the subject of future work.

## Figures and Tables

**Table 1:** Overview of the models established for MAE in the present work and for UAE in the previous work [18].

Output Modelled	MAE Model (Present Work)	UAE Model (Previous Work [18])
*t_b_* breaking time (min) (time when ultrasound generation switch from continuous to the discontinuous mode)	Not applicable	$t_{b}=V\left(\rho_{w}+X_{1}\left(\rho_{e}-\rho_{w}\right)\right)\left({Cp}_{w}+X_{1}\left({Cp}_{e}-{Cp}_{w}\right)\right)∆T\left(\frac{1}{{y\cdot X}_{3}}\right)$ where *V* is the solvent volume, *ρ_w_* and *ρ_e_* are water and ethanol densities, respectively, *Cp_w_* and *Cp_e_* are water and ethanol calorific capacities, respectively, and *y* is the efficiency of heat transmission to the solvent
*TP* or *AA* *TP* is the total polyphenol concentration (g.L^−1^ GAE) and *AA* is the antioxidant activity (mM TEAC): one equation of the same type was used for *TP* and for *AA*	$\left.\begin{array}{c} TP\\ or\\ AA \end{array}\right\}=\frac{1}{X_{2}}\left(A\left(1-e^{\left(-K_{1}t\right)}\right)+B\left(1-e^{\left(-K_{2}t\right)}\right)\right)$ where *t* is the extraction time; *A*, *B*, *K_1_* and *K_2_* are coefficients expressed as second order polynomial functions of input parameters (solvent composition *X_1_*, liquid/solid ratio *X_2_* and MW power density *X_3_*)	$\left.\begin{array}{c} TP\\ or\\ AA \end{array}\right\}=\frac{1}{X_{2}}\left(A\left(1-e^{\left(-K_{1}t\right)}\right)+\left\{\begin{array}{ll} B\left(1-e^{\left(-K_{2}t\right)}\right) & if\;t\leq t_{b}\\ C\left(1-e^{\left(-K_{3}\left(t-t_{b}\right)\right)}\right)+D & if\;t>t_{b} \end{array}\right.\right)$ with $D=B\left(1-e^{\left(-K_{2}t_{b}\right)}\right)$ where *t* is the extraction time; *t_b_* is the breaking time; *A*, *B*, *C*, *K_1_*, *K_2_* and *K_3_* are coefficients expressed as second order polynomial functions of input parameters (solvent composition *X_1_*, liquid/solid ratio *X_2_* and US power density *X_3_*)
*EC* Energy consumption (kWh)	$EC=P_{s}\cdot t+P_{c}\cdot t+P_{mw}\cdot t+E_{centr}$ where*P_s_* is the electric power consumed by the stirrer; *t* is the extraction time; *P_s_* is the electric power consumed by the water chiller; *P_mw_* is the power consumed for MW generation; *E_centr_* is the energy consumed for centrifugation	$EC={E_{centr}+P}_{s}\cdot t+\left\{\begin{array}{ll} P_{us}\cdot t & if\;t\leq t_{b}\\ P_{us}\cdot t_{b}+P_{us}^{*}\left(t-t_{b}\right) & if\;t>t_{b} \end{array}\right.$ where *E_centr_* is the energy consumed for centrifugation; *P_s_* is the electric power consumed by the stirrer; *t* is the extraction time; *t_b_* is the breaking time; *P_us_* is the power consumed for US generation in the continuous mode and $P_{us}^{*}$ the average power consumed for US generation in the discontinuous mode
*EI* 16 environmental impact categories with respective unit as defined in the ILCD Handbook, 2010 [23]: one equation of the same type was used for each of the 16 categories of impact	$EI=\overset{equipments}{\overbrace{\left(A_{eq}\cdot t+B_{eq}\right)}}+\overset{transports}{\overbrace{\left(A_{t}\cdot X_{1}+\frac{B_{t}}{X_{2}}\right)}}+\overset{raw\;materials}{\overbrace{\left(A_{rm}\cdot X_{1}+B_{rm}\right)}}+\overset{extraction}{\overbrace{\left(A_{ex}\cdot EC+B_{ex}\right)}}+\overset{end\;of\;life}{\overbrace{\frac{A_{eof}}{X_{2}}}}$ where *t* is the extraction time; *X_1_* and *X_2_* are the coded values for the solvent composition and the liquid/solid ratio, respectively; *A_eq_*, *B_eq_*, *A_t_*, *B_t_*, *A_rm_*, *B_rm_*, *A_ex_*, *B_ex_*, *A_eof_* are coefficients associated to each life cycle stage (equipment, transports, raw materials, extraction, end of life)	

**Table 2:** Predictions of the model for the experimental conditions and energy consumption enabling obtaining a maximal concentration of *TP* and the highest *AA* of the extracts.

Output	Maximum Output Value	Ethanol Content, %Vol.	Ratio L/S, mL.g^−1^	MW Power Density, W.L^−1^	Extraction Time, min	Energy Consumption, kWh
*TP*, g.L^−1^ GAE	3.7	65	5.0	1162	120	5.51
*AA*, mmol.L^−1^ TEAC	20.5	66	5.0	1198	120	5.59

**Table 3:** Model predictions for five scenarios at different constraints (target objective: *AA* of 16.6 mM TEAC).

	Constraint	Minimize *EC*	Minimize Time	Minimize Climate Change *EI*	Minimize Ethanol Content	Minimize MW Power Density
Input and Output Parameters						
Extraction time (*t*), min		19	17	20	120	120
Ethanol content (*X_1_*), %vol.		66	66	56	42	66
Ratio L/S (*X_2_*), mL.g^−1^		5	5	5	5	5
MW power density (*X_3_*), W.L^−1^		1300	1667	1663	1667	140
Energy consumption (*EC*), kWh		1.08	1.11	1.25	6.70	3.10

**Table 4:** Predictions of the models for three scenarios with different extraction processes, equipment and constraints (target objective: AA of 16.6 mM TEAC).

Process Input and Output Parameters	UAE	MAE (Constraint n°1)	MAE (Constraint n°2)
Extraction duration, min	120	19	30
Ethanol content, %vol.	65	66	65
Ratio L/S, mL.g^−1^	5	5	5
US power density, W.L^−1^	667	NA	NA
MW power density, W.L^−1^	NA	1300	667
Energy consumption, kWh	0.34	1.08	1.23

## Data Availability

The data are not available online.
